# Melatonin Regulates Differentiation of Sheep Brown Adipocyte Precursor Cells *Via* AMP-Activated Protein Kinase

**DOI:** 10.3389/fvets.2021.661773

**Published:** 2021-06-21

**Authors:** Xu-Yang Gao, Bu-Hao Deng, Xin-Rui Li, Yu Wang, Jian-Xin Zhang, Xiao-Yan Hao, Jun-Xing Zhao

**Affiliations:** College of Animal Science, Shanxi Agricultural University, Shanxi, China

**Keywords:** melatonin, brown adipogenesis, lambs, hypothermia, AMP-activated protein kinase

## Abstract

In sheep industry, hypothermia caused by insufficient brown adipose tissue (BAT) deposits is one of the major causes of lamb deaths. Enhancing the formation and function of BAT in neonatal lamb increases thermogenesis and hence reduces economic losses. The aim of the present study was to explore the effect and mechanism of melatonin on sheep brown adipocyte formation and function. Sheep brown adipocyte precursor cells (SBACs) isolated from perirenal BAT were treated with melatonin (1 and 10 nM). The SBACs subjected to melatonin exhibited a decreased proliferation ability, accompanied by down-regulated proliferating cell nuclear antigen, cyclin D1, and CDK4 protein contents in a melatonin dose-dependent manner. Melatonin promoted brown adipocyte formation and induced the expression of brown adipogenic markers, including *uncoupling protein 1* and *PR domain-containing 16* during differentiation of SBAC. Moreover, the AMP-activated protein kinase α1 (AMPKα1) activity was positively correlated with brown adipocyte formation potential. Importantly, melatonin effectively activated AMPKα1. Furthermore, promotional effects of melatonin were abolished by AMPKα1 knockout, suggesting the involvement of AMPKα1 in this process. Collectively, these results suggested that melatonin enhanced brown adipocyte formation in SBACs *in vitro* through activation of AMPKα1.

## Introduction

Mammalian brown adipose tissue (BAT) is responsible for metabolic heat generation through nonshivering thermogenesis, which is due to the high contents of uncoupling protein-1 (UCP1) and mitochondria (1). It is well-established that BAT plays an important role in mammalian thermoregulatory responses to a cold environment. In sheep industry, hypothermia is one of the major causes of lamb deaths (2, 3). Considering that heat production by BAT accounts for approximately half of the heat needed by newborn lambs to maintain central temperature (4), the BAT is essential for the survival of newborns, especially for those born in a cold winter. Therefore, exploring the underlying mechanisms regulating BAT development and function is important for sheep production and welfare.

The AMP-activated protein kinase (AMPK) is a conserved heterotrimeric enzyme containing catalytic α, regulatory β, and γ subunits (5). Besides serving as central regulator of multiple metabolic pathways, AMPK is indispensable for early embryonic development and growth and mediates differentiation of various cell types (6). Most recent studies prove that AMPK activity is necessary for development and maintenance of BAT (7), which may epigenetically regulate *Prdm16* and *Idh2* transcription (8, 9).

Melatonin (*N*-acetyl-5-methoxytryptamine) is an ancient multifunctional molecule ubiquitously presented in plants and animals (10). In mammals, circulating melatonin is largely synthesized and released to the blood by the pineal gland during nighttime. Meanwhile, melatonin is also produced in several tissues and organs, including the gut, ovary, testes, and retina, for local utilization (through autocrine and paracrine actions) (11). The physiological functions of melatonin, including circadian and seasonal rhythms, antioxidation, antitumor, tissue repair and regeneration, immunomodulation, and metabolism, have been reviewed and discussed by different laboratories (12, 13).

Recent studies demonstrate the regulatory role of melatonin on BAT development. For instance, melatonin administration effectively promoted BAT growth, increased BAT weight, and enhanced its activity in rodent and human (14, 15). Moreover, maternal melatonin suppression during gestation decreased BAT mass of newborn lambs (16). Interestingly, melatonin effectively activates AMPK during mouse C3H10T1/2 cell osteogenic differentiation (17). Until now, whether melatonin directly regulates BAT development in lambs remains undefined. The objective of this study was to investigate the effects of melatonin on sheep brown adipocyte formation and to reveal the underlying mechanisms of this effect.

## Materials and Methods

### Brown Adipocyte Precursor Cell Isolation and Differentiation

Unless otherwise indicated, all chemicals were purchased from Sigma-Aldrich (St. Louis, MO, USA). The sheep brown adipocyte precursor cell (SBAC) isolation was performed according to a previous published protocol (18) with a minor modification. Briefly, ewe at day 90 of gestation was euthanized, and dissected fetal perirenal BAT (from twin premature fetal lambs) was isolated with scissors and pooled. Then, the BAT was disinfected with 75% ethanol and rinsed thoroughly with phosphate-buffered saline (PBS) containing 1% PSN antibiotic (penicillin/streptomycin/neomycin; Sigma-Aldrich). Disinfected BAT pieces were minced with scissors and digested in a 50 mL Falcon tube containing 40 mL Dulbecco modified eagle medium/F12 (DMEM/F12; Hyclone, Waltham, MA, USA) with 5% fetal bovine serum (FBS), 1 mg/mL collagenase A, 0.05% hyaluronidase, and 1% PSN antibiotic by gentle agitation at 37°C for 40 min. Following digestion, the suspension was sequentially filtered through 100- and 70-μm cell strainers (Corning, NY, USA), and filtrates were centrifuged at 800 × *g* for 10 min at room temperature. The progenitors were resuspended in DMEM/F12 medium with 10% FBS and 1% PSN antibiotic for further use.

The brown adipogenesis induction was performed by following the established protocol in our laboratory (8). Briefly, the SBACs were induced at 100% confluence by addition of brown adipogenic medium containing 0.5 mM isobutyl-1-methylxanthine (IBMX), 1 μM dexamethasone, 1 nM triiodothyronine (T3), 125 nM indomethacin, and 1 mg/mL insulin. After 48 h, cells were switched to maintain medium containing 1 nM T3 and 1 mg/mL insulin, and medium was refreshed every 2 days until day 6.

### Wound Healing Scratch Assay

The SBACs achieving 100% confluence were scored with a sterile pipette tip to leave a scratch (~0.5 mm) and washed with DMEM for three times. After that, cells were subjected to melatonin (1 and 10 nM) or vehicle, and the wound closure was tracked via DMi8 microscope (Leica, Germany) at 12, 24, and 36 h after the scratching.

### EdU Staining

Cell proliferation ability was evaluated using Cell-Light™ EdU DNA cell proliferation kit (C10310-1; RiboBio, Guangzhou, China) by following the manufacturer's instructions. Briefly, the SBACs at logarithmic phase were treated with melatonin (1 and 10 nM) or vehicle for 24 h. After exposing to 50 μM EdU for 2 h, cells were fixed with 4% paraformaldehyde, permeabilized by 0.5% Triton X-100, and incubated with 1 × Apollo reaction cocktail. The EdU-labeled cells were captured under DMi8 fluorescence microscope (Leica).

### Protein Preparation and Western Blotting

Cells grown on 12-well plates were harvested and lysed in 100 μL of an ice-cold lysis buffer. Then, lysates were centrifuged for 15 min at 12,000 × *g*, 4°C, and soluble protein was subjected to sodium dodecyl sulfate-polyacrylamide gel electrophoresis for protein separation (4°C, 100 V for 2 h). The separated proteins were transferred to nitrocellulose (NC) membrane (4°C, 100 V for 2 h). After blocking with 5% skim milk power (Sangon Biotech Co., Ltd., Shanghai, China), the NC membranes were incubated with primary antibodies (1:1,000, 4°C, overnight), followed by secondary antibody at room temperate for 1 h. The immunoblotting bands were scanned and analyzed using Odyssey Infrared Imaging System (LI-COR Biosciences, Lincoln, NE, USA). Band density was normalized to the β-actin content.

### Antibodies and Plasmid

The antibodies against proliferating cell nuclear antigen (PCNA, bs-0754R), cyclin D1 (bs-0623R), cyclin-dependent kinase 4 (CDK4, bs-0633R), β-actin (bs-0061R), UCP1 (bs-1925R), peroxisome proliferator-activated receptor γ (PPARγ, bs0530R), CCAAT/enhancer-binding protein β (C/EBPβ, bs-1396R), and AMPKα1 (bs-10344R) were purchased from Biosynthesis Biotechnology Co., Ltd. (Beijing, China). p-AMPKα (2,535), acetyl-CoA carboxylase (ACC, 3662), p-ACC (3,661), and anti-rabbit fluoresce secondary antibody (4,412) were purchased from Cell Signaling Technology (Danvers, MA, USA). The anti-PR-domain-containing 16 (PRDM16, PA5-20872) was from Thermo Fisher Scientific (Waltham, MA, USA). The anti-coenzyme Q-cytochrome c reductase (complex III, 21705-1-AP) and ATP synthase (complex V, 14676-1-AP) were purchased from Sanying Biotechnology Co., Ltd. (Wuhan, China). The goat anti-mouse secondary antibody (926-68070) and anti-rabbit secondary antibody (926-32211) were sourced from LI-COR Biosciences. Scrambled CRISPR/Cas and AMPKα1-CRISPR/Cas9 (plasmid 79004) were obtained from Addgene Inc (Cambridge, MA, USA).

### Oil-Red O Staining

Differentiated cells were washed twice in ice-cold PBS and fixed with 10% formaldehyde for 10 min at room temperature. Subsequently, cells were washed with both PBS and 60% isopropyl alcohol and incubated with 0.2% (vol/vol) oil-red O solution (Sigma Chemical Co., St. Louis, MO, USA) at room temperature for 30 min. After that, cells were rinsed with 60% isopropanol for 15 s and washed twice with PBS. Images were taken under a DMi8 microscope (Leica Microsystems, Wetzlar, Germany). The dye was solubilized with isopropanol, and absorbance was obtained using Synergy H1 Multi-Mode Reader (BioTek, Winooski, VT, USA).

### RNA Preparation and Real-Time Quantitative Polymerase Chain Reaction

Total RNA was extracted using RNeasy Mini Kit (QIAGEN, Germany) by following the supplier's protocol. Concentration and the integrity of isolated RNA were evaluated by NanoDrop ND-2000 instrument (Nanodrop Instruments, Delaware, USA). Cells were counted blindly using Cell Counting Chamber (0650030, Marienfeld, Germany), and a total of 5 ×10^5^ were seeded into 12 wells plate. A total of 1 μg of RNA from each sample was reverse transcribed into cDNA using a reverse transcription kit (Takara Co., Ltd., Dalian, China). Quantitative real-time polymerase chain reaction (qPCR) was performed using the CFX reverse transcriptase (RT)-PCR detection system (Bio-Rad, Hercules, CA, USA) and an SYBR Green RT-PCR kit (Takara Co., Ltd.) according to the manufacturer's instructions. Relative mRNA content was normalized to the 18S rRNA content, and the 2^−Δ*ΔCt*^ method was carried to determine relative changes in gene expression. The primer sequences are shown in Table 1.

**Table 1:** Primer sequences for real-time PCR.

**Name**	**Sequence (5^**′**^-3^**′**^)**	**Length (bp)**
*UCP1*	TTGCTTCTCTCAGGATCGGC	133
	GTGGGTTGCCCAATGAACAC	
*PRDM16*	GCCTGTTTCTCTTCTGTCCCC	130
	GCCAACAGGACGGTGTTATTT	
*Cidea*	TGCATCCTCCAAGCGTTTCT	121
	CCTCCTGTTCAGTCCACACC	
*COX7A1*	CGGTGCAACAGACAACATCC	129
	GTCCCGCAGACTTCTTGGTT	
*PGC-1α*	TGTCGGATGCTTGCTTGAGT	109
	TACGGTTGTAACGCAGGACCT
*Cytochrome C*	CAGGGTTGTCCTAAACAGGAA	89
	CTGAGTTGGCAAAAGCACGA	
*18s rRNA*	*CTCTAGATAACCTCGGGCCG*	209
	GTCGGGAGTGGGTAATTTGC	

### Immunocytochemical Staining

The SBACs grown on coverslips were differentiated into brown adipocytes with addition of melatonin (1 and 10 nM) or vehicle. After washing with cold PBS, cells were fixed with cold methanol, permeabilized with 0.25% Triton X-100, and blocked with 3% bovine serum albumin. Subsequently, cells were incubated with UCP1 antibody (1:200) at 4°C overnight, followed by fluorescent secondary antibody incubation. Finally, cells were mounted, and images were captured with DMi8 microscope (Leica, Germany).

### Plasmid Transfection

Plasmid was electroporated in 100 μL nucleocuvettes using the Lonza 4D Nucleofector System (4D-Nucleofector™ core unit together with Primary Cell Optimization 4D-Nucleofector™ X Kit) (Cat: V4XP-9096; Lonza, Basel, Switzerland) according to the manufacturer's instructions.

### Statistical Analysis

All analyses were conducted using GraphPad Prism 7 software package (Monrovia, CA, USA). Data were expressed as mean ± standard error of the mean (SEM), whereas homogeneity of variance and normality were evaluated using the Brown-Forsythe test and the Shapiro-Wilk. One-way analysis of variance or Student *t*-test with a two-tailed distribution was used where appropriate, and Duncan multiple-range test was employed for *post hoc*. *P* < 0.05 was considered as statistically significant for all data.

## Results

### Melatonin Inhibited Sheep Brown Precursor Cell Proliferation

**Figure 1:**
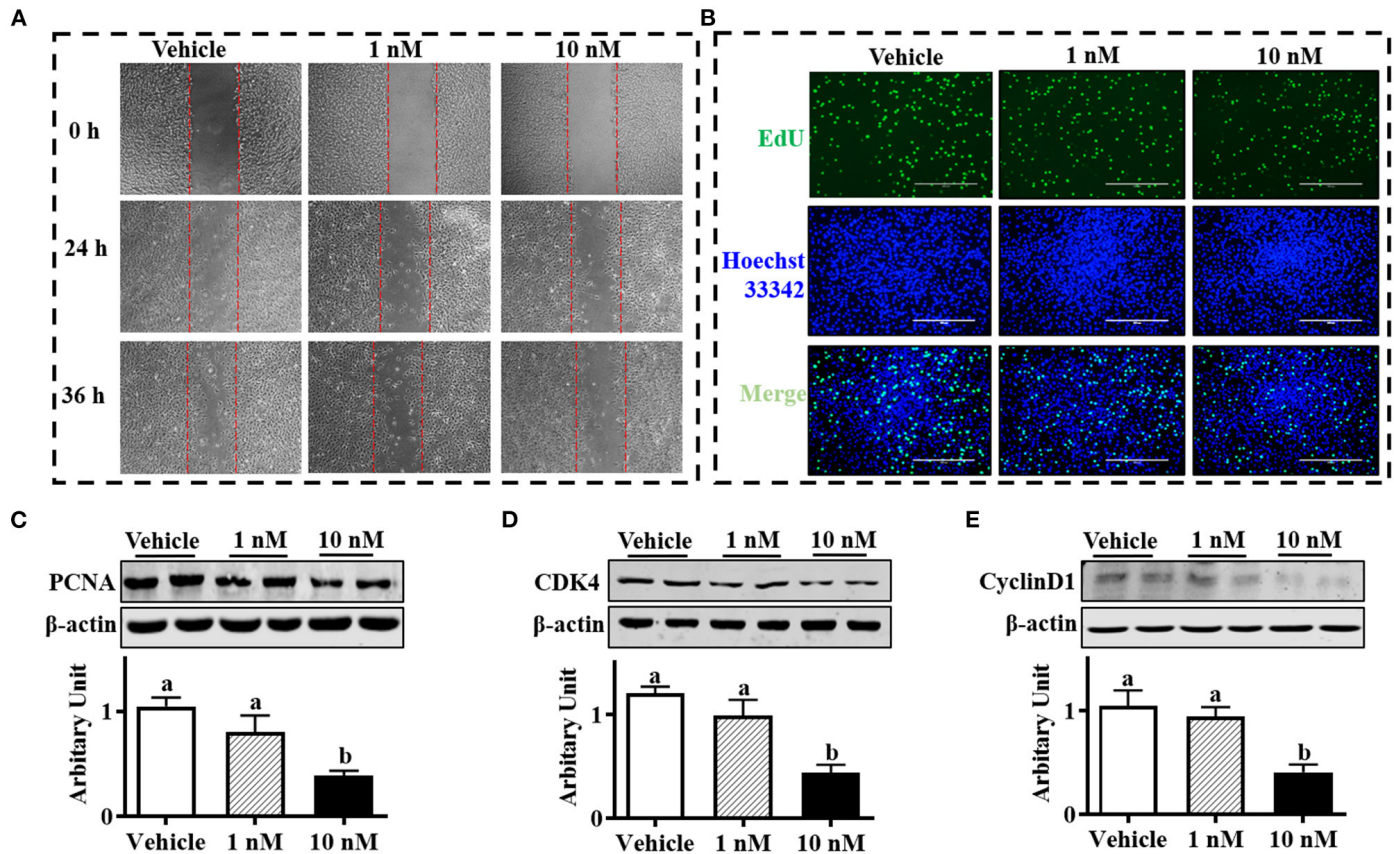
Melatonin inhibited sheep brown precursor cell proliferation. Isolated cells were treated with melatonin (1 and 10 nM) or vehicle for 24 h. **(A)** Wound healing scratch assay. **(B)** EDU staining. **(C–E)** PCNA, cyclin D1, and CDK4 protein abundances analyzed by Western blotting (*n* = 6, mean ± SEM; different letters mean significant difference).

As shown in Figure 1A, the SBACs subjected to melatonin for 24 h exhibited decreased proliferation ability. The EdU staining is widely used to track proliferating cells in multiple biological systems, and more EdU staining means more proliferating cells. The results indicated that EdU-positive cell numbers were declined in a melatonin dose-dependent manner, and 1 nM melatonin was sufficient to decrease positive cell number (Figure 1B). Accordingly, precursor cells that received 10 nM melatonin showed decreased PCNA protein abundance (Figure 1C, *P* <.05). As expected, 10 nM melatonin reduced both cyclin D1 and CDK4 protein abundances (Figures 1D,E; *P* < 0.01)

### Melatonin Enhanced Brown Adipocyte Formation

**Figure 2:**
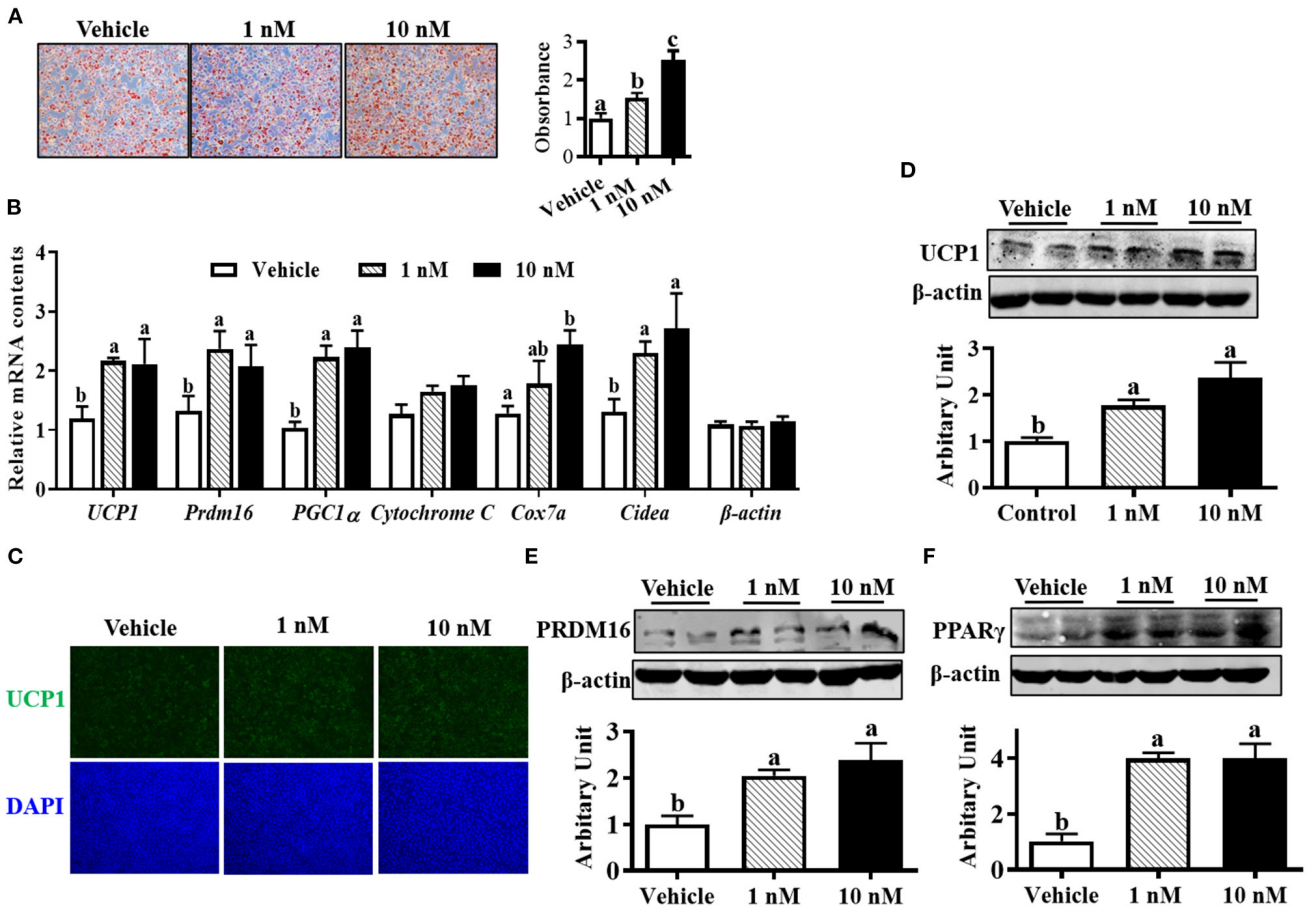
Melatonin promoted brown adipocyte formation. **(A)** Cells were induced to brown adipogenesis for 6 days with or without melatonin (1 and 10 nM), and adipocytes were visualized by oil-red O staining (100 × magnification). **(B)** Relative mRNA expression of brown adipogenic markers. **(C)** Immunocytochemistry staining of UCP1 after 6 days of differentiation. **(D–F)** Protein abundances of UCP1, PRDM16 and PPARγ (*n* = 6, mean ± SEM; different letters mean significant difference).

Oil-red O staining suggested that both 1 and 10 nM melatonin increased lipid accumulation after 6 days of differentiation (Figure 2A, *P* < 0.05). Moreover, the mRNA expressions of brown adipogenic markers, including *UCP1, Prdm16, cell death-inducing DNA fragmentation factor-*α*-like effector A (Cidea*), *Cox7a*, and *peroxisome proliferator-activated receptor-*γ *coactivator* (*PGC1*α), were up-regulated in 10 nM melatonin-treated cells (Figure 2B, *P* < 0.05). Brown adipocytes were quantified by UCP1 immunocytochemical staining. As shown in Figure 2C, the number of UCP1-positive cells was elevated by melatonin addition, consistent with a greater protein level of UCP1 (Figure 2D, *P* < 0.05). Moreover, both 1 and 10 nM melatonin effectively increased PRDM16 and PPARγ protein contents (Figures 2E,F; *P* < 0.05), which further confirmed the enhanced brown adipogenesis differentiation by melatonin.

### Melatonin Activated AMPK in Sheep Brown Precursor Cell

**Figure 3:**
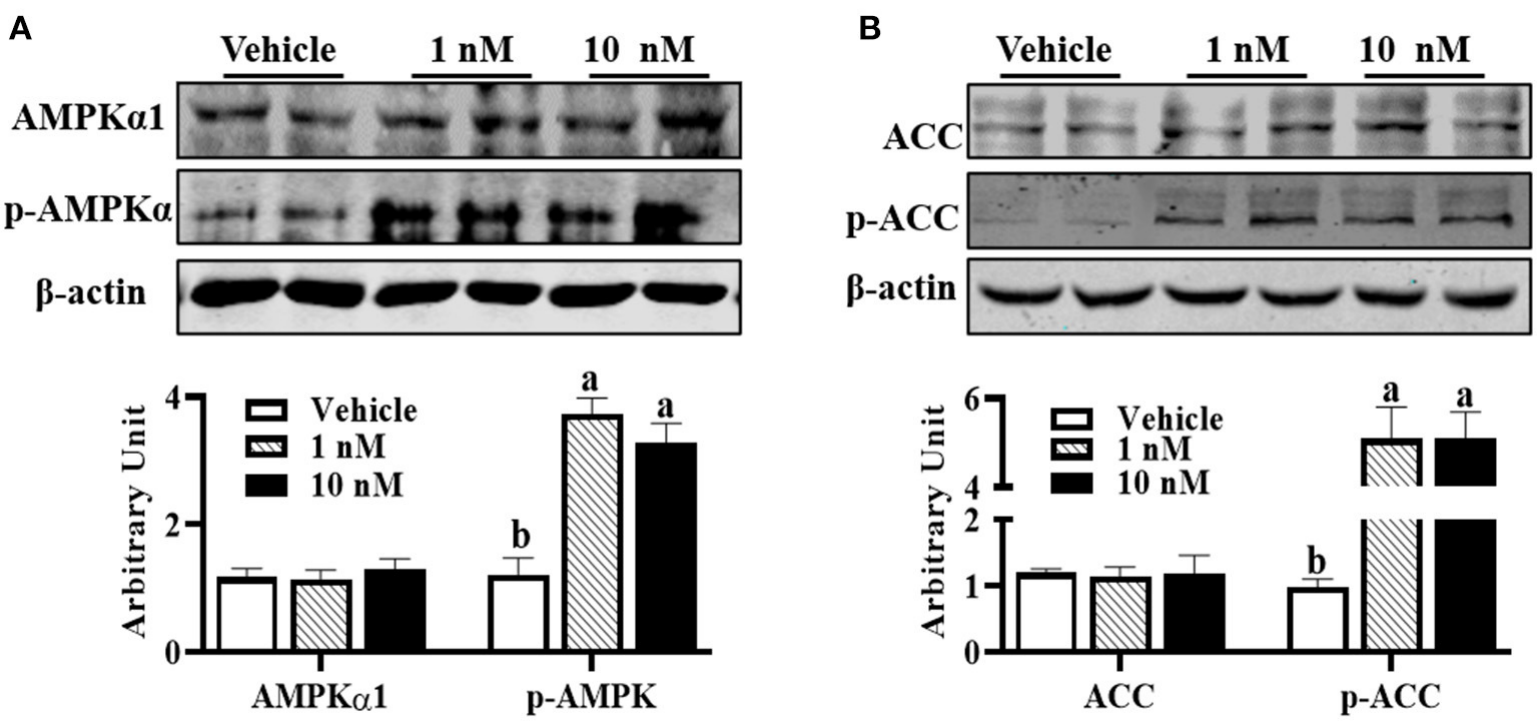
Melatonin activated AMPK during precursor cell differentiation. Isolated precursor cells were treated with indicated concentrations of melatonin for 24 h, and Western blot analysis was performed to analyze **(A)** AMPKα1, p-AMPKα, **(B)** ACC, and p-ACC protein abundances (*n* = 6, mean ± SEM; different letters mean significant difference).

Compared with control cells, although AMPKα1 (pThr172) protein content was not affected in melatonin-treated groups, 1 nM melatonin was sufficient to increase p-AMPKα1 protein level (Figure 3A, *P* < 0.01). Supportively, increased p-ACC protein contents were observed in melatonin-treated cells (Figure 3B, *P* < 0.01), which further indicated the activation of AMPKα1.

### Necessity of AMPKα1 in Sheep Brown Adipogenesis

**Figure 4:**
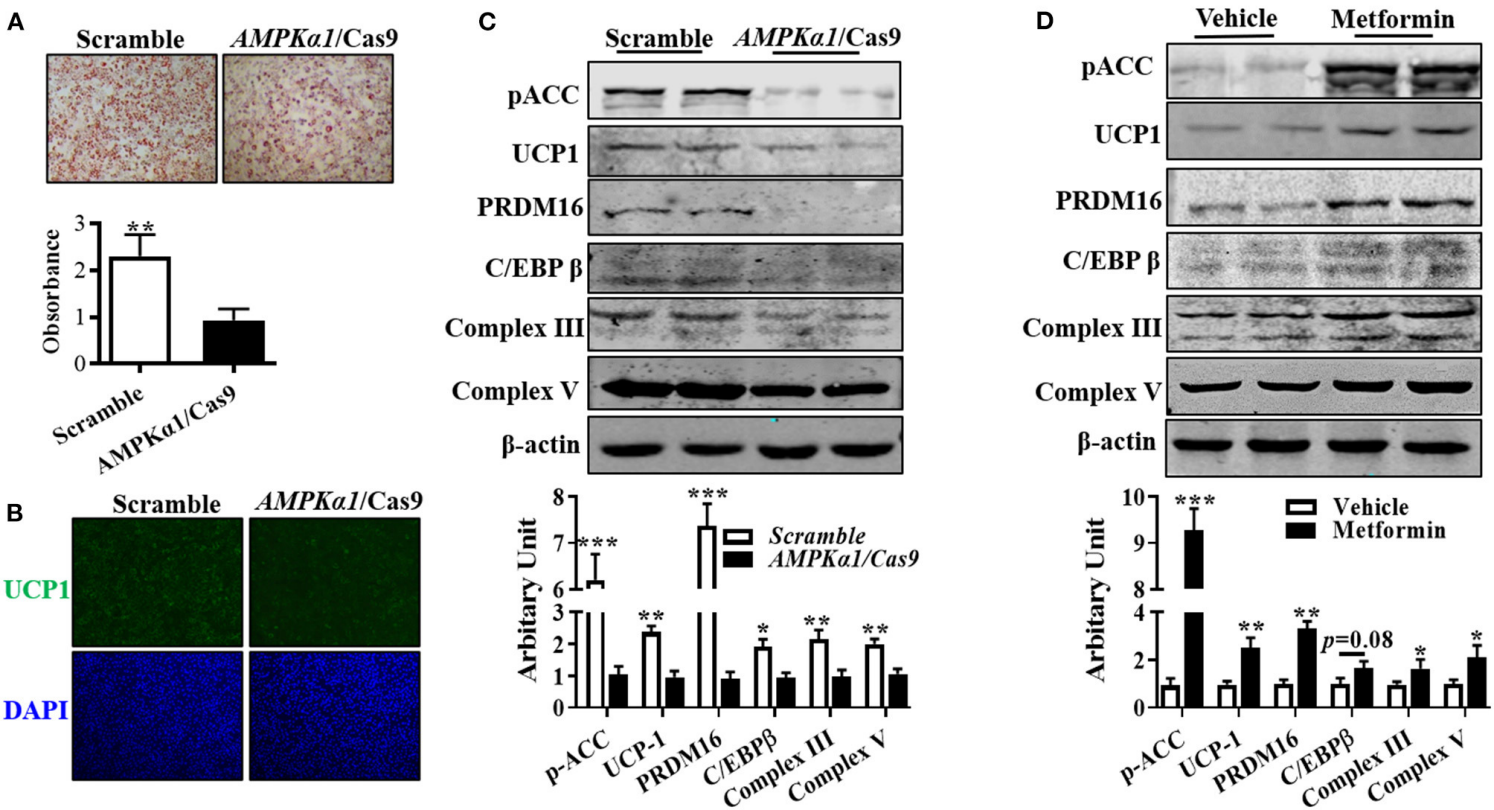
AMPKα1 activity was necessary for brown adipocyte formation. Precursor cells were transfected with either scrambled Cas9 or *AMPK*α*1* CRISPR/Cas9 and induced brown adipogenesis. **(A,B)** Adipocytes were visualized by oil-red O staining **(A)** and immunocytochemistry staining of UCP1 after 6 days of differentiation **(B)**. **(C,D)** Protein abundances of p-ACC, UCP1, PRDM16, C/EBPβ, complex III, and complex V. Cells were treated with metformin (2 mM) and vehicle, and protein contents of p-ACC, UCP1, PRDM16, C/EBPβ, complex III, and complex V were analyzed using Western blotting (*n* = 6, mean ± SEM; *P < 0.05, **P < 0.01, ***P < 0.001).

Compared with wild-type cells, lipid droplet accumulation in AMPKα1 knockout (KO) brown adipocytes was decreased (Figure 4A). Immunocytochemically, transfecting precursor cells with *AMPK*α*1*/Cas9 knocking out plasmid remarkably reduced UCP1 contents in brown adipocytes (Figure 4B), which was further confirmed by immunoblotting, showing that UCP1 protein level was attenuated (Figure 4C, *P* < 0.01). As shown in Figure 4C, AMPKα1 KO also attenuated C/EBPβ (*P* < 0.05) and PRDM16 (*P* < 0.01) abundances, as well as complex III (*P* < 0.01) and complex V (*P* < 0.01) protein contents. Furthermore, activation of AMPK by metformin effectively elevated the aforementioned proteins level (Figure 4D).

### Melatonin Regulated Sheep Brown Precursor Cell Differentiation via AMPKα1

**Figure 5:**
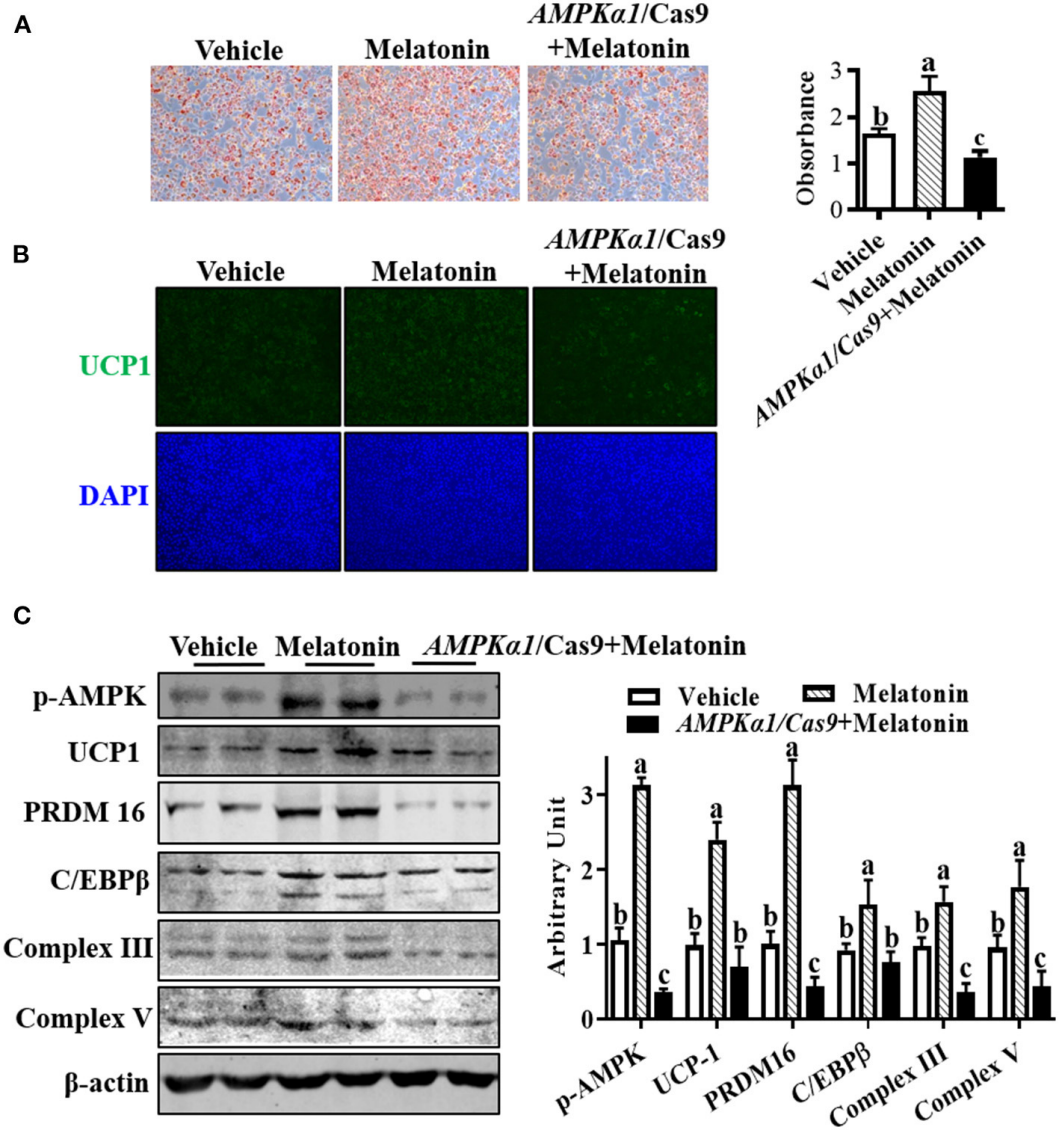
Melatonin facilitated brown adipogenesis through AMPKα1. Precursor cells were treated with 10 nM melatonin or vehicle; simultaneously, *AMPK*α*1* knockout cells were subjected to 10 nM melatonin. **(A, B)** Brown adipogenesis was evaluated by oil-red O staining **(A)** and immunocytochemistry staining of UCP1 after 6 days of differentiation **(B)**. Data showed that the number of brown adipocytes was compromised when *AMPK*α*1* was knockout. **(C)** The p-ACC, UCP1, PRDM16, C/EBPβ, complex III, and complex V protein contents among different groups (*n* = 6, mean ± SEM; different letters mean significant difference).

To investigate whether melatonin regulated SBAC differentiation through AMPKα1, we used oil-red O staining to assess lipid accumulation in brown adipocytes. As Figure 5A shows, the effect of melatonin on lipid accumulation was compromised when AMPKα1 was KO. Meanwhile, the effects observed in melatonin-treated cells were absent in AMPKα1 KO cells, detected by UCP1 staining (Figure 5B). As shown in Figure 5C, the immunoblotting analyses suggested that AMPKα1 KO attenuated the regulatory effect of melatonin on key brown adipogenic-related protein contents, including UCP1, PRDM16, C/EBPβ, complex III, and complex V.

## Discussion

In mammals, adipocytes are generally divided into white, brown, and beige (brite) adipocyte based on morphological and biological function. The white adipocytes serve as the principal site for energy storage in the form of triacylglycerol, whereas brown adipocytes metabolize fatty acids and glucose for heat production, and the inducible beige adipocytes also possess thermogenic capability (19). In lambs, the ability to utilize nonshivering thermogenesis in BAT during the first 12 h after birth is vital to prevent hypothermia in the perinatal period (20). Therefore, insufficient BAT deposits and inability to metabolize BAT, combined with cold extrauterine environment, are the major causes of lamb death (20). Lambs as opposed to rodents are born with mature BAT, and thus, sheep maternal manipulation is feasible to increase neonate BAT mass and function. Until now, experimental evidence supporting potential functions of melatonin in fetal BAT development is limited. Previous studies indicate that sheep maternal melatonin during gestation effectively accrues BAT mass and stimulates the thermogenic activity in the neonatal lambs (16). Moreover, maternal melatonin implants from day 100 of gestation increases BAT deposition (21), and underlying mechanisms remain unclear.

The major deposit of BAT in fetal lambs is the perirenal adipose tissue, which increases rapidly between days 70 and 120 of gestation (22). Generally, many steps of brown adipogenesis can be recapitulated through *in vitro* differentiation of precursor cells. Therefore, perirenal brown adipocyte precursor cells isolated from day 90 of gestation were used to evaluate the effect of melatonin on sheep brown adipogenesis. Despite that numerous previous studies demonstrate that melatonin affects cell proliferation, the effect of suppression or stimulation varies, depending on the cell type examined and melatonin's concentration (23). In the present study, melatonin exhibited an antiproliferative effect on sheep brown preadipocytes, which might be attributed to melatonin's effects on PCNA, a standard marker of cell proliferation (24). Similarly, decreased PCNA contents are observed in melatonin-treated prostate cancer (25). The mechanisms of how melatonin affected PCNA expression need to be further studied. The cyclin D1/cdk4 complex has been strongly implicated in the control of cell proliferation (26). In human keratinocytes, down-regulation of cyclin D1 is important for cell cycle exit and initiation of differentiation (27). Therefore, by down-regulation of cdk4 and cyclin D1, melatonin exerted an antiproliferative effect on sheep brown preadipocytes, in line with a previous report showing that melatonin inhibits human osteosarcoma cell proliferation through the down-regulation of cyclin D1 and cdk4 (28).

As mentioned previously, maternal melatonin promoted BAT development in the neonatal lambs. In mammals, melatonin acts through two G-protein–coupled membrane receptor subtypes, named MT1 and MT2 (10). Notably, a functional melatonin receptor has been identified in fetal lambs BAT (29), suggesting that melatonin might directly regulate brown adipogenesis. In the present study, melatonin was found to effectively increase the number of brown adipocytes, which was accompanied by elevated PRDM16 protein. Considering that PRDM16 was one of the key transcriptional factors involved in brown adipogenesis (30), the results suggested that melatonin directly regulated adipogenesis of sheep brown preadipocytes. The UCP1 is the thermogenic effector of BAT, which uncouples the substrates of oxidation and ATP formation during the process of mitochondrial electron transport (31). Therefore, the elevated UCP1 abundance further suggested the regulatory effect of melatonin on brown adipogenesis. Indeed, binding of melatonin to receptor has been proved to cause a reduction of intracellular cAMP, which further decreases protein kinase A activity and subsequently up-regulates the expression of UCP1(32). The brown adipogenesis is a tightly regulated process, which involves preadipocyte proliferation and adipocyte differentiation. After clonal expansion that occurs in the early phase of adipogenesis, cells will permanently exit from the cell cycle and undergo terminal differentiation. Although proliferation ability was declined in melatonin-treated cells, the differentiation ability was enhanced by melatonin, which may eventually lead to BAT growth.

The next question was how melatonin regulated brown adipocyte formation. Previous studies demonstrate that melatonin up-regulates the activity of AMPK in various tissues and organs of rodents (17, 33). Moreover, melatonin enhances osteogenic differentiation of mesenchymal stem cells through AMPK activation (34). Considering that AMPK is a highly conserved eukaryotic protein complex, we asked if melatonin-activated AMPKα1 in sheep brown preadipocytes. As expected, the AMPKα1 activity was elevated by melatonin, which was confirmed by an increased p-ACC abundance.

Previous studies demonstrate that maternal cold exposure or l-arginine administration during late pregnancy increases BAT mass and stimulates the thermogenic activity in the neonatal lambs (35, 36). Moreover, the AMPKα1 activity is essential for the epigenetic control of BAT development in mice (9). Coincidently, cold exposure causes a progressive increased AMPKα1 activity in BAT (37), whereas l-arginine enhances AMPK expression and activity (38). Therefore, we hypothesized that AMPKα1 activity was positively related to BAT development in lambs. In the present study, *AMPK*α*1* ablation in SBACs attenuated brown adipogenesis, whereas AMPK activation by metformin enhanced brown adipogenic potential. It is well-established that both mitochondrial complex III and complex V play a pivotal role in electron transfer and oxidative phosphorylation, and their abundances reflect mitochondrial function (39). Thus, our data further suggested the regulatory role of melatonin on mitochondrial function and activity, similar with previous study showing that melatonin enhances mice neural stem cell differentiation by increasing mitochondrial function (40).

To the best of our knowledge, the exact regulation mechanisms of melatonin on BAT development and function remain unclear. To further elucidate whether melatonin regulated brown adipogenesis *via* interaction with AMPKα1 in these precursors, we treated *AMPK*α*1* KO cells with melatonin. The compromised effects of melatonin on brown adipogenesis in *AMPK*α*1* KO cells suggested that the regulatory effects of melatonin might be, if not all, through interaction with AMPKα1. Given the fact that AMPKα1 epigenetically regulates *prdm16* transcription (9) and promotes mitochondrial biogenesis and function by phosphorylating DNMT1, RBBP7, and HAT1 (41), our data suggested that AMPKα1 built a bridge between melatonin and sheep brown adipocyte formation. Furthermore, our results provided direct evidence that melatonin regulated sheep brown fat cell formation and function and thus might provide a strategy for maintaining or improving body temperature of newborn lamb.

## Data Availability Statement

The original contributions presented in the study are included in the article/supplementary material, further inquiries can be directed to the corresponding author.

## Ethics Statement

The animal study was reviewed and approved by Institutional Animal Care and Use Committee of Shanxi Agricultural University.

## Author Contributions

J-XZhao and X-YG conceived and designed the experiments. X-YG, B-HD, and X-RL performed the experiments. J-XZhan and YW analyzed the data. J-XZhao and X-YH drafted and revised the manuscript. All authors contributed to the article and approved the submitted version.

## Conflict of Interest

The authors declare that the research was conducted in the absence of any commercial or financial relationships that could be construed as a potential conflict of interest.

## Abbreviations

BAT: brown adipose tissue
PCNA: proliferation cell nuclear antigen
CDK4: cyclin-dependent kinase 4
AMPKα1: 5′-adenosine monophosphate-activated protein kinase
p-AMPKα1: phospho-5′-adenosine monophosphate-activated protein kinase
UCP1: uncoupling protein 1
PRDM16: PR domain containing 16
C/EBPβ: CCAAT-enhancer–binding protein beta
complex III: coenzyme Q-cytochrome c reductase
complex V: ATP synthase
KO: knockout
PGC1α: peroxisome proliferator-activated receptor-γ coactivator
Cidea: cell death–inducing DNA fragmentation factor-α-like effector A
Cox7a: cytochrome c oxidase polypeptide 7a
ACC: acetyl-CoA carboxylase
p-ACC: phospho-acetyl-CoA carboxylase.
